# “Nunca mais o Brasil sem nós”: povos indígenas no *Censo Demográfico 2022*

**DOI:** 10.1590/0102-311XPT232223

**Published:** 2024-05-17

**Authors:** Alessandra Traldi Simoni, Bruno Nogueira Guimarães, Ricardo Ventura Santos

**Affiliations:** 1 Universidade Estadual de Campinas, Campinas, Brasil.; 2 Universidade Federal do Rio de Janeiro, Rio de Janeiro, Brasil.; 3 Centro de Apoio Operacional às Promotorias de Justiça para Apoio Comunitário, Inclusão e Mobilização Sociais, Ministério Público do Estado de Minas Gerais, Belo Horizonte, Brasil.; 4 Escola Nacional de Saúde Pública Sergio Arouca, Fundação Oswaldo Cruz, Rio de Janeiro, Brasil.; 5 Museu Nacional, Universidade Federal do Rio de Janeiro, Rio de Janeiro, Brasil.

No dia 7 de agosto de 2023, aconteceu, em Belém, no Pará, Brasil, o evento *O Brasil Indígena: Uma Nova Foto da População Indígena*, organizado pelo Instituto Brasileiro de Geografia e Estatística (IBGE). Na ocasião, foram apresentados os primeiros resultados do *Censo Demográfico 2022* acerca da população indígena. Foi divulgada a impactante cifra de 1.693.535 pessoas declaradas indígenas, o que representa um aumento de 88% em relação ao censo anterior, de 2010, que contabilizou aproximadamente 890 mil. No evento, Sônia Guajajara, a Ministra de Estado dos Povos Indígenas, afirmou “*nunca mais o Brasil sem nós*”, o que aponta para a centralidade dos dados censitários para fins de inclusão e visibilidade.

Na trajetória dos recenseamentos nacionais, que teve início em 1872, a população indígena foi captada de variadas formas, mas, na maior parte dos levantamentos, esteve subsumida à categoria “parda” ou variantes. Somente a partir do censo de 1991 a categoria “indígena” passou a ser captada no quesito “cor ou raça”. Desse modo, em tempos recentes, o IBGE levantou de forma contínua dados sobre a população indígena em quatro recenseamentos, sendo eles: 1991, 2000, 2010 e 2022. É uma série histórica relativamente curta, mas de enorme significado e implicação no tocante à visibilidade demográfica desse segmento da população.

A captação de dados sobre a população indígena nos recenseamentos nacionais, assim como a presença de registros em outras fontes de estatísticas públicas, como é o caso dos sistemas de informação em saúde, associa-se proximamente a mudanças implementadas a partir da promulgação da *Constituição Federal de 1988*, em que são “*reconhecidos aos povos indígenas sua organização social, costumes, línguas, crenças e tradições*” (art. 231). Dessa forma, visibilizar os povos indígenas nas estatísticas públicas faz parte desse processo histórico e sócio-político nacional, que também se associa à ampliação do foco acerca das populações originárias no plano internacional e na América Latina, com um esforço dos institutos de estatística em promover mudanças metodológicas nos censos, buscando retratar a diversidade étnico-racial ^1^^,^^2^.

Quanto ao detalhamento das características étnicas e socioculturais, foi somente a partir do censo de 2010 que passaram a ser coletados dados como pertencimento étnico específico e línguas indígenas faladas nos domicílios. No censo de 2022, os temas investigados se expandiram, com a criação de um questionário específico sobre as comunidades indígenas. Houve esforços também no sentido de ampliar a “base territorial” para a identificação dos domicílios indígenas, com a atualização das informações sobre as Terras Indígenas de 2010 para 2022 e a identificação das chamadas “localidades indígenas” no território nacional, por meio de uma “cartografia colaborativa” ^3^, com informações providas por organizações indígenas, instituições governamentais e organizações não governamentais (ONG) indigenistas. Para o planejamento e execução do censo de 2022, constituiu-se uma ampla rede de atores sociais e institucionais, considerando também situações específicas, como é o caso de povos indígenas de recente contato e em situação de refúgio. Destaca-se, também, a participação indígena desde a etapa de planejamento (envolvendo lideranças e organizações indígenas), assim como na execução (com guias, tradutores, recenseadores e supervisores indígenas). Esses elementos certamente tiveram influência sobre os resultados do recenseamento, inclusive por potencialmente favorecer uma maior capilaridade e cobertura das operações conduzidas pelo IBGE.

Ao se abordar os resultados do censo de 2022 acerca da população indígena no Brasil, com seu montante superior a 1,5 milhão, é imprescindível considerar os quantitativos registrados nos recenseamentos anteriores de uma perspectiva comparativa: 294.148 em 1991; 734.127 em 2000; e 896.917 em 2010. Desse modo, a população indígena em 2022 é quase seis vezes maior que na década de 1990 e quase o dobro em relação a 2010. Se, em 2010, a população indígena constituía 0,4% da população nacional, passou a 0,8% em 2022. Apesar disso, o Brasil continua a ser um dos países nas Américas com a menor proporção de sua população constituída por indígenas.

Essas variações ao longo dos censos, como destacam diversas análises produzidas pelo IBGE e por estudos da comunidade científica, não pode ser interpretada com derivando unicamente das inter-relações entre fatores demográficos, como natalidade e mortalidade, tampouco devido à migração ^2^^,^^4^^,^^5^. As mudanças nos montantes da população indígena se vinculam a questões metodológicas, tanto no tocante à cobertura e capilaridade dos levantamentos em âmbito territorial como decorrente dos conteúdos das perguntas e de uma maior quantidade de pessoas se reconhecendo como indígenas.

Com efeito, as perguntas de captação da população indígena nos censos de 2010 e 2022 são cruciais para compreender o aumento observado no quantitativo de indígenas no país. Desde longa data se discute a adequação da pergunta sobre “cor ou raça” para fins da captação dos quantitativos acerca da população indígena ^1^^,^^2^^,^^5^. Considerando essa questão, foi criada, em 2010, uma “pergunta de cobertura” que deveria ser aplicada apenas em recortes territoriais específicos, naquele caso apenas nos setores censitários localizados nas Terras Indígenas ^6^. Assim, se o entrevistado se declarasse em outra categoria que não indígena (branca, preta, amarela ou parda), era feita uma pergunta adicional - “você se considera indígena?”. Considerou-se então população indígena a soma das pessoas que se declararam e consideraram indígenas, essa última correspondendo a 9% do total em 2010. No censo de 2022, a abertura dessa questão foi ampliada para todas as localidades indígenas, dentro e fora das Terras Indígenas, em áreas rurais e urbanas, perfazendo 27% do total de 1,6 milhão de indígenas ^3^. Essa constatação de que aproximadamente 1/4 da população indígena captada não deriva diretamente da categoria “indígena” na pergunta acerca de “cor ou raça” demandará reflexões ao longo dos próximos anos.

A metodologia implementada pelo IBGE no censo de 2022, que ampliou a aplicação da pergunta de cobertura “se considera”, contribuiu para um aumento expressivo de declarações indígenas em localidades e regiões nas quais havia acontecido baixa captação anteriormente ^3^. Isso pode ser exemplificado pelas variações observadas em capitais da Região Norte, que é aquela do país com o maior contingente indígena. Aconteceram aumentos em praticamente todas as capitais da região, com exceção de Belém. Um exemplo marcante é aquele de Manaus, capital do Amazonas, com uma população indígena de pouco mais de 70 mil em 2022, em comparação a 4 mil em 2010. Também em larga medida devido à presença indígena fora de Terras Indígenas, o Estado da Bahia passou a ser o segundo, após o Amazonas, com maior volume de população indígena. Merece destaque, também, o aumento da população indígena fora de Terras Indígenas em municípios próximos a elas, como Tefé (Amazonas), Altamira (Pará), Pesqueira (Pernambuco) e Dourados (Mato Grosso do Sul).

A partir dos resultados já divulgados para os indígenas residentes fora de Terras Indígenas, serão geradas informações com desdobramentos significativos para fins de políticas públicas em saúde e em vários outros campos, especialmente em áreas urbanas ^3^. É conhecido que diversos centros urbanos têm áreas com forte presença indígena, como o Parque das Tribos em Manaus, a comunidade de Paraisópolis em São Paulo e a Aldeia Marçal de Souza em Campo Grande (Mato Grosso do Sul). Muitos outros exemplos poderiam ser mencionados em cidades de vários portes, que apontam para a importância das informações a serem divulgadas, considerando a análise do pertencimento étnico, perfis socioeconômicos e condições de moradia.

Até o momento, poucos recortes dos resultados censitários de 2022 sobre a população indígena foram divulgados, como a população total e sua distribuição em alguns níveis geográficos (como Estado, municípios, Terra Indígena). Informações acerca de etnias, línguas faladas, composição etária e sexo estão sendo gradativamente divulgadas nos próximos meses. Em 2010, os resultados do IBGE indicaram a existência de 305 povos indígenas e 272 línguas indígenas faladas no país. Os perfis que emergirão a partir dos resultados do censo de 2022 delinearão novos cenários acerca da sociodiversidade indígena no país.

Os resultados serão fundamentais também na ampliação de discussões para a promoção dos direitos dos povos indígenas em escala internacional, como é o caso da *Agenda 2030* e *Consenso de Montevidéu*. Vale citar também a importância das informações do censo de 2022 para a implementação de iniciativas como a pioneira *Resolução sobre a Saúde dos Povos Indígenas*^7^ da Organização Mundial da Saúde (OMS), de 2023, que, entre muitas questões, aborda a importância de fontes e sistemas de informação para análises de iniquidades na saúde dos povos indígenas.

Nas investigações sobre os determinantes sociais da saúde, com foco na dimensão étnico-racial, os resultados do censo de 2022 têm muitas implicações para os estudos sobre desigualdades no país. Os dados censitários são utilizados como denominadores nos mais diversos indicadores relacionados ao adoecimento e morte de populações. Os dados do censo de 2010 resultaram em inúmeras investigações que apontaram para as iniquidades socioeconômica e de saúde da população indígena em comparação com a população brasileira em geral ^8^. Uma profunda discussão, e muita cautela, deverão estar presentes nos estudos em saúde coletiva a serem desenvolvidos a partir dos dados do censo de 2022, uma vez que, em alguns contextos, houve um aumento significativo da população indígena, como no caso de Manaus, que é o município que passa a ter a maior população indígena no país. Nesse caso, as análises temporais dos indicadores precisarão considerar o aumento de mais de 66 mil pessoas, o que terá um efeito direto nos denominadores de diversos indicadores, com a possibilidade de redução de taxas. Devido às implicações, tais resultados deverão ser analisados por meio de perspectivas que considerem que as variações resultam também de mudanças em padrões de captação de dados e de reconhecimento étnico, sendo necessário, portanto, acionar chaves explicativas para além da variação populacional decorrente de fatores demográficos.

Nesse sentido, a divulgação dos resultados dos censos demográficos sempre leva a debates. No caso da população indígena, discussões sobre a captação de dados em censos anteriores promoveram mudanças na metodologia de coleta ^3^^,^^4^^,^^6^. Sendo uma pesquisa tão ampla e abrangente, e considerando que muitas comunidades indígenas se localizam em regiões de difícil acesso, questionamentos quanto aos dados coletados precisam ser ponderados. Ao mesmo tempo, os censos demográficos são hoje a mais importante fonte de dados sobre os povos indígenas no Brasil e constituem referência internacional na captação de dados desse segmento populacional ^2^^,^^5^.

Nessa fase que se abre de divulgação dos resultados do censo de 2022, uma abordagem importante será estimular que comunidades, pesquisadores e gestores indígenas se envolvam na análise e produção de interpretações a partir dos dados censitários. A pauta da importância das estatísticas públicas nos debates e as iniciativas dos movimentos etnopolíticos indígenas têm sido crescentemente enfatizadas em vários países do mundo, inclusive no Brasil. Um exemplo disso foi o protagonismo indígena na produção e divulgação de dados durante a pandemia de COVID-19 ^9^, em um período quando as posturas do então governo eram francamente anti-indígenas. Destaca-se, também, a participação indígena no censo de 2022 ^3^ e recentes iniciativas pós-censitárias, como atividades de capacitação de pesquisadores e gestores indígenas acerca dos dados censitários, em iniciativas da Associação Brasileira de Estudos Populacionais, em parceria com o IBGE e o Fundo de População das Nações Unidas (UNFPA) no Brasil.

Durante o evento em Belém, citado anteriormente, a centralidade dos dados censitários da população indígena para as políticas públicas foi enfatizada por Joenia Wapichana, presidente da Fundação Nacional dos Povos Indígenas (FUNAI) e Ricardo Weibe Tapeba, secretário de Saúde Indígena do Ministério da Saúde. Refletindo sobre as perspectivas do movimento etnopolítico indígena acerca da importância dos resultados do IBGE, ambos indicaram como a “visibilização” da população indígena é a chave para o planejamento e a implementação de políticas públicas nas mais diversas áreas por parte do governo. Nesse sentido, que os dados censitários sejam um instrumento para a efetivação do “*nunca mais o Brasil sem nós*” de Sonia Guajajara!
